# Failure Factors to Reach the Blind End Using a Short-Type Single-Balloon Enteroscope for ERCP with Roux-en-Y Reconstruction: A Multicenter Retrospective Study

**DOI:** 10.1155/2019/3536487

**Published:** 2019-05-05

**Authors:** Yusuke Kawaguchi, Hiroshi Yamauchi, Mitsuhiro Kida, Kosuke Okuwaki, Tomohisa Iwai, Kazuho Uehara, Rikiya Hasegawa, Hiroshi Imaizumi, Kiyonori Kobayashi, Wasaburo Koizumi

**Affiliations:** ^1^Department of Gastroenterology, Kitasato University Medical Center, 6-100 Arai, Kitamoto-shi, Saitama 364-8501, Japan; ^2^Department of Gastroenterology, Kitasato University Hospital, 1-15-1 Kitasato, Minami-ku, Sagamihara, Kanagawa 252-0375, Japan; ^3^Department of Research and Development Center for New Medical Frontiers, Kitasato University East Hospital, 2-1-1 Asamizodai, Minami-ku, Sagamihara, Kanagawa 252-0380, Japan

## Abstract

**Background:**

Failure factors in reaching the blind end (papillae of Vater, bilioenteric anastomosis) during short-type single-balloon enteroscope-assisted endoscopic retrograde cholangiopancreatography (sSBE-assisted ERCP) in patients with Roux-en-Y (R-Y) reconstruction remain to be evaluated.

**Aims:**

We investigated the failure factors in such patients.

**Methods:**

We retrospectively studied 253 initial sessions of sSBE-assisted ERCP at three endoscopy centers from April 2008 through September 2017, examining failure factors and complications associated with scope insertion in patients with R-Y reconstruction.

**Results:**

R-Y reconstruction was performed in 157 patients (with gastrectomy: 122 patients; without gastrectomy plus bilioenteric anastomosis: 35 patients). R-Y without gastrectomy (*p* = 0.001; odds ratio (OR), 5.73; 95% confidence interval (CI), 2.07 to 16.01) and the presence of peritoneal dissemination (*p* = 0.021; OR, 4.71; 95% CI, 1.27 to 17.54) were significant failure factors. Insufficient sSBE length was the cause of failure in 17 (11%) of the 157 patients, and 13 (76%) of the 17 patients were with R-Y without gastrectomy. In cases of insufficient short-type length, using a long-type SBE significantly increased the success rate (*p* = 0.002). Gastrointestinal stenosis was a significant failure factor (*p* = 0.011) in patients with peritoneal dissemination. Perforation occurred in 2 patients who responded to conservative treatment.

**Conclusions:**

Failure factors during sSBE-assisted ERCP were R-Y without gastrectomy and the presence of peritoneal dissemination.

## 1. Introduction

Recently, endoscopic retrograde cholangiopancreatography (ERCP) has been widely performed using balloon enteroscopes in patients with Roux-en-Y (R-Y) reconstruction, and good results have been reported [1–13]. However, ERCP in such patients is technically challenging, and the first problem is whether an endoscope can reach the blind end, defined as the papilla of Vater or the bilioenteric anastomosis [13].

Balloon enteroscopes can be divided into two types: long-type enteroscopes developed to examine and treat the small bowel and short-type enteroscopes developed to perform not only gastrointestinal examination but also ERCP in patients with surgically altered anatomy. Most conventional devices for ERCP cannot be used due to the length of the scope when ERCP is performed using a long-type enteroscope. Therefore, adequate treatment cannot be performed. Conversely, when ERCP is performed using a short-type enteroscope, most conventional devices for ERCP can be used because of the shorter length. However, the success rate of reaching the blind end seems to be decreased. The success rate of reaching the blind end and the convenience of treatment have been reported to be satisfactory for short-type enteroscopes [3–9]. However, in some patients, the blind end can only be reached using a long-type enteroscope [14]. The failure factors in reaching the blind end with a short-type enteroscope remain to be fully investigated.

Therefore, we retrospectively studied factors related to the inability to reach the blind end when ERCP was performed using a short-type single-balloon enteroscope (sSBE) in patients with R-Y reconstruction.

## 2. Patients and Methods

### 2.1. Patients

From April 2008 through March 2017, a total of 435 sessions of sSBE-assisted ERCP were performed in patients with surgically altered anatomy in Kitasato University Hospital, Kitasato University East Hospital, and Kitasato University Medical Center. We studied 253 initial sessions of sSBE-assisted ERCP using the endoscopic databases of these facilities. The indication of sSBE-assisted ERCP was for patients with symptomatic pancreatobiliary diseases (e.g., obstructive jaundice, cholangitis, and pancreatitis due to the bile duct stone), same indication for ERCP for patients with normal gastrointestinal anatomy. sSBE-assisted ERCP was contraindicated for patients with American Society of Anesthesiologists (ASA) classification score 5, clear evidence of gastrointestinal stenosis, or intestinal obstruction evaluated through preoperative computed tomography (CT). All patients provided written informed consent before the procedure.

### 2.2. Methods

We studied age, sex, type of R-Y reconstruction (with gastrectomy: distal or total gastrectomy; without gastrectomy: bile duct resection with bilioenteric anastomosis), reason for surgery (malignant disease or benign disease), indications for ERCP (malignant disease or benign disease), body mass index (BMI) (<18.5 or ≥18.5), ASA classification score (≤2 or ≥3), presence or absence of peritoneal dissemination, past history of postoperative intestinal obstruction, presence or absence of splenectomy, number of abdominal operations (1 time or ≥2 times), whether surgical records were obtained before examination, the experience level of the endoscopist (trainer or trainee), and whether or not passive bending and high force transmission was equipped. All cases of peritoneal dissemination were histologically proven to be peritoneal seeding. Endoscopists who had performed less than 30 sessions of SBE-assisted ERCP were defined as trainees, and those who had performed 30 or more sessions were defined as trainers.

The primary outcome measure was the failure factors in reaching the blind end using an sSBE in patients with R-Y reconstruction. The secondary outcome measures were the reasons for failure to reach the blind end and the success rate of reaching the blind end using a long-type SBE (lSBE) in patients in whom failure to reach the blind end using an sSBE was caused by an insufficient length of the scope and complications associated with sSBE insertion. Pancreatitis associated with endoscope insertion was defined as pancreatitis affecting only the body and tail of the pancreas [15, 16]. The severity of complications was evaluated according to the severity grading system of the American Society for Gastrointestinal Endoscopy [17]. This study was approved by the institutional review board of each participating hospital.

### 2.3. Endoscopic Procedures

Patients were sedated with pethidine (50 mg) and midazolam (3 to 10 mg), and vital signs were intermittently monitored during all procedures. Propofol was used if necessary. Carbon dioxide insufflation was used in all procedures. The enteroscope was inserted with the patient in the prone position, and abdominal compression was applied manually if in-depth insertion was technically difficult.

Examinations were performed by 14 endoscopists who met the following conditions: (1) The total colonoscopy success rate in the most recent 100 sessions was at least 95%. (2) The rate of successful intubation of the bile duct or the pancreatic duct during ERCP in patients with normal gastrointestinal anatomy was at least 95%.

### 2.4. Endoscopes and Instruments

We used four models of enteroscopes: SIF-Y0004, SIF-Y0004-V01, and SIF-Y0015, which were prototype enteroscopes, and SIF-H290S, which was a commercially available model (Olympus Medical Systems, Tokyo, Japan). All enteroscopes had a working length of 1520 mm, a working channel diameter of 3.2 mm, and a distal end outer diameter of 9.2 mm. With the exception of SIF-Y0004, all enteroscopes had passive bending and high force transmission functions. A sliding tube with a working length of 880 mm was used (ST-SB1S, Olympus Medical Systems). A tip cap (D-201-10704, Olympus Medical Systems) was used in all patients.

### 2.5. Statistical Analysis

Categorical variables were analyzed using Fisher's exact test. Continuous variables were examined using Student's *t*-test or Welch's *t*-test. Multivariate analysis was performed using logistic regression analysis. Variables with *p* values of <0.1 on univariate analysis were included in the multivariate analysis; *p* values of <0.05 were considered to indicate statistical significance. Statistical analysis was performed with the use of BellCurve for Excel version 2.00 (Social Survey Research Information Co. Ltd., Japan).

## 3. Results

**Figure 1:**
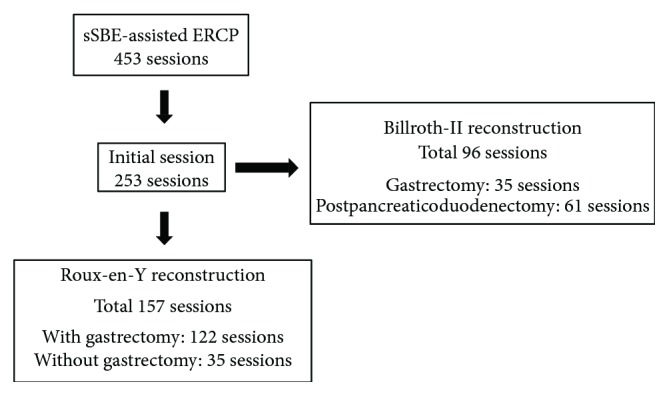
A total of 157 initial sessions of sSBE-assisted ERCP in the patient with R-Y reconstruction were evaluated in this study.

A total of 157 patients with R-Y reconstruction were enrolled in this study (Figure 1). The study group comprised 121 men and 36 women with a mean (±standard deviation) age of 72.5 (±9.9) years. R-Y with gastrectomy was performed in 122 patients, and R-Y without gastrectomy was performed in 35 patients. The detailed characteristics of the patients are shown in Table 1.

### 3.1. Failure Factors to Reach the Blind End (Table 2)

**Figure 2:**
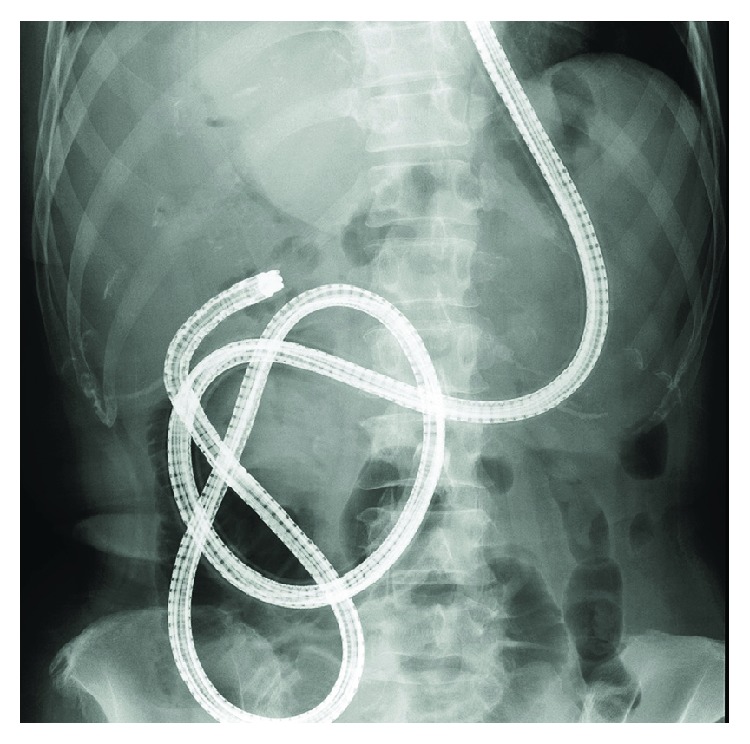
Roux-en-Y reconstruction without gastrectomy. A short-type SBE could reach the blind end.

**Figure 3:**
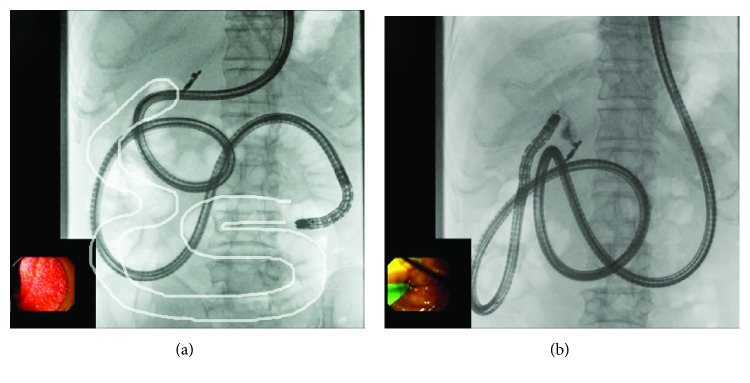
Roux-en-Y reconstruction without gastrectomy. A short-type SBE could not reach the blind end. (a) A short-type SBE could not reach the blind end because the length of the enteroscope was too short to adequately shorten the intestine. The white line indicates the course of the intestine up to the choledochojejunal anastomosis as confirmed during insufflation on radiography. (b) After switching to a long-type SBE, the intestine was successfully shortened. The blind end was reached.

Univariate analysis showed that age (*p* = 0.089), female sex (*p* = 0.085), R-Y without gastrectomy (*p* = 0.001), presence of peritoneal dissemination (*p* = 0.035), and not obtaining surgical records (*p* = 0.027) had *p* values of ≤0.1. Multivariate analysis showed that R-Y without gastrectomy (*p* = 0.001; odds ratio (OR), 5.73; 95% confidence interval (CI), 2.07-16.01) (Figures 2 and 3) and the presence of peritoneal dissemination (*p* = 0.021; OR, 4.71; 95% CI, 1.27-17.54) were significant factors for failure to reach the blind end.

### 3.2. Causes of Failure to Reach the Blind End (Table 3)

The causes of failure to reach the blind end in the 29 patients who underwent R-Y reconstruction were an insufficient sSBE length in 17 patients (59%), malignant gastrointestinal stenosis caused by peritoneal dissemination in 5 patients (17%), and others in 7 patients (24%).

### 3.3. The Success Rate of Reaching the Blind End Using an lSBE (Table 4)

Insertion of an lSBE was attempted in all patients in whom an sSBE could not reach the blind end because of an insufficient sSBE length. The overall success rate of reaching the blind end was 82% (14/17). In the patients who underwent R-Y without gastrectomy, the blind end was successfully reached with an sSBE alone in 60% of the patients and with an sSBE+lSBE in 91% of the patients. The use of an lSBE significantly increased the success rate of reaching the blind end (*p* = 0.002).

### 3.4. Complications

Endoscopic insertion was associated with perforation in 2 patients (1.3%). All cases of perforation responded to conservative medical treatment, and the severity was moderate. There were no other complications, such as pancreatitis or pneumonia.

## 4. Discussion

The purpose of our study was to investigate the failure factors in reaching the blind end using an sSBE in patients with R-Y reconstruction. In our study, failure to reach the blind end was associated with patients who had not had gastrectomy and the presence of peritoneal dissemination.

In clinical practice, R-Y without gastrectomy is the longest distance to the blind end in countries and regions where R-Y gastric bypass is rarely performed in obese patients. Difficulty in reaching the blind end in R-Y is often encountered since the distance to the blind end is longer than that of other reconstructions. The biggest drawback of using an sSBE is that the shorter scope length may decrease the success rate of reaching the blind end [3]. In our study, an insufficient sSBE length was responsible for the failure to reach the blind end in 17 (59%) of the 29 patients in whom reaching the blind end was unsuccessful (Table 3). In terms of the types of reconstruction, 13 (76%) of the 17 patients underwent R-Y without gastrectomy. An insufficient sSBE length was the cause of failure to reach the blind end in 4 of the 122 patients (3.3%) with R-Y with gastrectomy and 13 of the 35 patients (37%) with R-Y without gastrectomy. The insufficient enteroscope length, which was a concern when an sSBE was used, was considered to be acceptable in patients with R-Y with gastrectomy. However, the main reason for not reaching the blind end was attributed to the insufficient sSBE length in patients with R-Y without gastrectomy. In such patients, the use of an lSBE significantly increased the success rate of reaching the blind end (*p* = 0.002). These results indicate that using an lSBE from the initial examination is a feasible option with R-Y without gastrectomy, although treatment options may be limited. However, the usefulness of an lSBE with a channel diameter of 3.2 mm has recently been reported [18]. If many types of devices are developed for a long type, an lSBE may become the first choice enteroscope for endoscopic insertion and treatment in patients with R-Y without gastrectomy. And the use of a short-type double-balloon enteroscope (sDBE) may be one of the option in patients with R-Y without gastrectomy, because ERCP using an sDBE in patients with R-Y reconstructions showed a high success rate [9].

**Figure 4:**
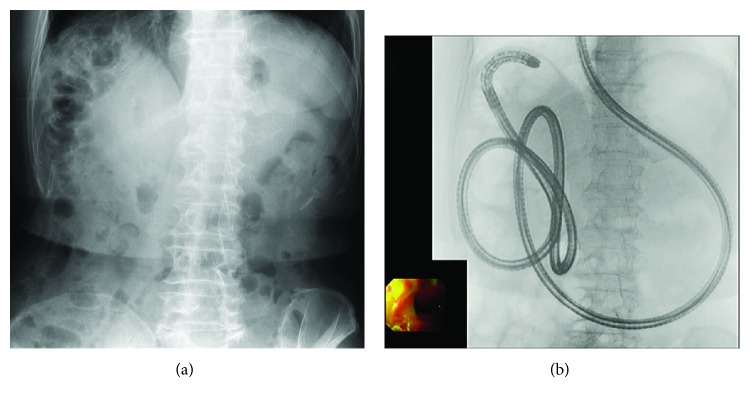
Right hepatic lobectomy+Roux-en-Y reconstruction without gastrectomy. (a) Owing to the effect of right hepatic lobectomy, the intestine was displaced under the diaphragm, similar to patients with Chilaiditi's syndrome. (b) A short-type SBE could not reach the blind end, but a long-type SBE could reach it.

Furthermore, in patients with hepatectomy, the intestines may enter the space created by the hepatectomy, often making enteroscope insertion difficult. In particular, reaching the blind end will be more difficult in patients with right hepatic lobectomy because the intestines may become displaced under the diaphragm, similar to patients with Chilaiditi's syndrome, and often make manual compression ineffective (Figure 4). In our study, the success rate of reaching the blind end in patients with R-Y without gastrectomy was 56% (5/9) with hepatectomy, 62% (16/26) without hepatectomy, 33% (2/6) with right hepatic lobectomy, and 66% (19/29) without right hepatic lobectomy. The success rate of reaching the blind end was lower in patients with right hepatic lobectomy, but not significantly. Because we studied only 35 patients, further studies with larger numbers of patients are necessary.

**Figure 5:**
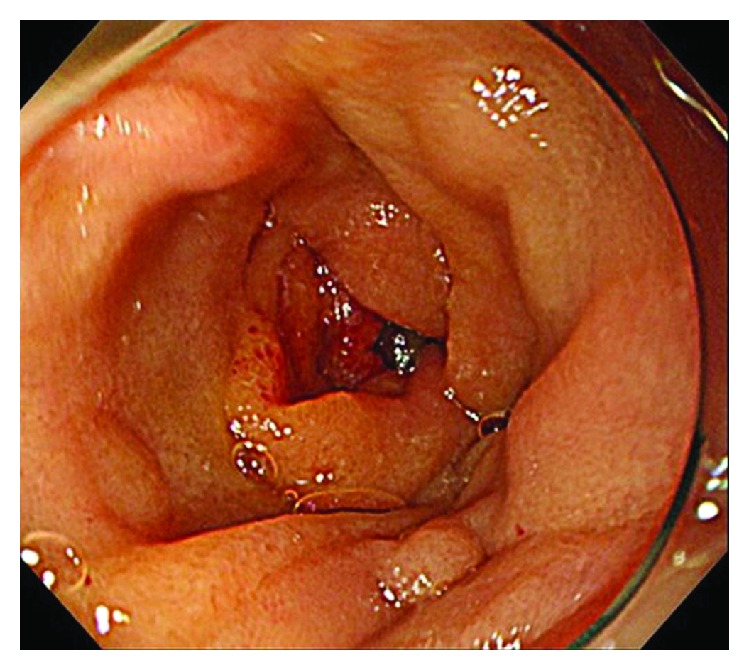
Malignant jejunal stricture caused by peritoneal dissemination after surgery for gastric cancer.

Peritoneal dissemination is a factor associated with failure to reach the blind end in patients with R-Y reconstruction. In patients with peritoneal dissemination, the success rate of reaching the blind end was 17 % (1/6) with a malignant gastrointestinal stricture and 89% (8/9) without a malignant gastrointestinal stricture. The presence of malignant gastrointestinal strictures (Figure 5) significantly decreased the success rate of reaching the blind end (*p* = 0.011). Patients with peritoneal dissemination may have a latent gastrointestinal stricture even if no clear evidence of gastrointestinal strictures or ileuses was seen on preoperative CT images. In patients with a gastrointestinal stricture, the cause of failure to reaching the blind end includes the inability to physically pass an enteroscope or an overtube through the stricture and the fact that endoscopic procedures were severely restricted even if an enteroscope or overtube could be inserted.

**Figure 6:**
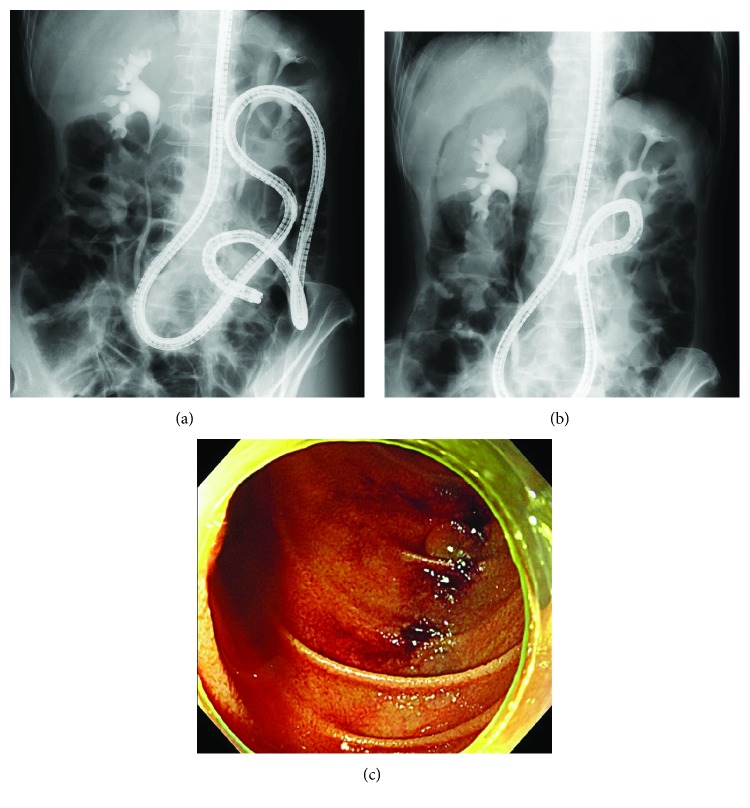
Perforation in a patient with peritoneal dissemination of gastric cancer. (a) An sSBE was inserted into the Treitz ligament, and a urethral stricture caused by peritoneal dissemination was found after contrast-enhanced CT. (b) When the intestine was shortened and an sSBE was inserted into the horizontal portion of the duodenum, free air was found around the right kidney. (c) When an sSBE advanced to the descending portion of the duodenum, 3 lacerations were found in the intestine contralateral to the papilla.

Complications associated with endoscopic insertion in this study included perforations in 2 patients (1.3%). In one patient, perforation was caused by a laceration that occurred when the sSBE was withdrawn. The other patient (Figure 6) had peritoneal dissemination and was found to have free air on radiography when the intestine was shortened at the Treitz ligament; the enteroscope was therefore inserted into the horizontal portion of the duodenum. Three lacerations were found in the descending portion of the duodenum that could not be reached by an sSBE at the time of perforation. The lacerations were apparently caused by insufflation or intestinal shortening. Caution is required in patients with peritoneal dissemination because excessive insufflation and intestinal shortening are risk factors for perforations.

In both described cases, the cause of free air was a laceration of the duodenal wall. Although the air penetrated a laceration of the duodenal wall, no leakage of contrast media was observed. Therefore, the clip was not used for suturing the duodenal wall, and we chose conservative treatment. The severity of perforation was moderate in both patients and responded to conservative medical treatment. There were no serious complications. Therefore, the insertion of an sSBE is considered to be safe.

This study has several limitations. First, it was retrospective. Second, R-Y gastric bypass was not included as it is rarely performed in Japan. Third, it was reported that the success rate of balloon enteroscope-assisted ERCP is depended on the length of the blind loop [19]. However, we could not get the length of the blind loop, because it was not described in the surgical record in almost all cases. However, our study was a multicenter study and a large number of initial sessions of sSBE-assisted ERCP were analyzed, excluding repeated procedures.

In conclusion, factors related to failing to reach the blind end during sSBE-assisted ERCP were R-Y without gastrectomy and the presence of peritoneal dissemination. In patient with R-Y without gastrectomy, using an lSBE may become one of the most effective treatment strategies. In addition, patients with peritoneal dissemination may have latent gastrointestinal strictures not evident on preoperative CT images.

## Figures and Tables

**Table 1:** Patients' characteristics.

	R-Y with gastrectomy *N* = 122, *n* (%)	R-Y without gastrectomy *N* = 35, *n* (%)	Total (*n* = 157)
Age, mean ± SD (years)	74.0 ± 7.9	70.0 ± 13.9	
Sex, *n* (%)			
Male	101 (83)	20 (57)	121 (77)
Female	21 (17)	15 (43)	36 (23)
Reasons for surgery, *n* (%)			
Ulcer of the upper GI tract	3 (2.5)	0 (0)	3 (2)
Malignancy of the upper GI tract	117 (96)	3 (8)	120 (76)
Malignancy of the biliary tract	0 (0)	16 (46)	16 (10)
Biliopancreatic congenital abnormalities	0 (0)	9 (26)	9 (6)
Others	2 (1.5)	7 (20)	9 (6)
Reason for ERCP, *n* (%)			
Bile duct stones	83 (68)	20 (57)	103 (65.5)
Carcinoma of the pancreas	5 (4)	1 (3)	6 (4)
Malignancy of the biliary tract	14 (11.5)	0 (0)	14 (9)
IPMN	3 (2.5)	0 (0)	3 (2)
Metastasis of L/n in porta hepatis	8 (6.5)	0 (0)	8 (5)
Stricture of the anastomotic site	0 (0)	12 (34)	12 (7.5)
Others	9 (7.5)	2 (6)	11 (7)
BMI, *n* (%)			
<18.5	48 (39)	5 (14)	53 (34)
≥18.5	74 (61)	30 (86)	104 (66)
ASA score, *n* (%)			
≤2	82 (67)	28 (80)	110 (70)
≥3	40 (33)	7 (20)	47 (30)
Peritoneal dissemination, *n* (%)			
Present	14 (11)	1 (3)	15 (10)
Absent	108 (89)	34 (97)	142 (90)
Number of abdominal operations, *n* (%)			
1 time	86 (70)	25 (71)	111 (71)
≥2 times	36 (30)	10 (29)	46 (29)
Surgical records, *n* (%)			
Available	80 (66)	18 (51)	98 (62)
Not available	42 (34)	17 (49)	59 (38)

AOSC: acute obstructive suppurative cholangitis; ASA: American Society of Anesthesiologists; BMI: body mass index; CBD: common bile duct; ERCP: endoscopic retrograde cholangiopancreatography; GI: gastrointestinal; IPMN: intraductal papillary mucinous neoplasm; IPNB: intraductal papillary neoplasm of the bile duct; R-Y: Roux-en-Y; SD: standard deviation.

**Table 2:** Failure factors for reaching the blind end.

Background factors	Reached *N* = 128	Not reached *N* = 29	Rate of reaching the blind end	Univariate analysis *p* value	Multivariate analysis *p* value	Odds ratio (95% CI)
Age, mean ± SD (years)	73.1 ± 8.1	69.8 ± 12.5	—	0.089	0.734	
Sex						
Male	102	19	84%	0.085	0.57	
Female	26	10	72%			
Types of R-Y reconstruction						
With gastrectomy	107	15	88%	0.001	0.001	5.73 (2.07-16.01)
Without gastrectomy	21	14	60%			
Reason for surgery						
Malignant disease	114	23	83%	0.134		
Benign disease	14	6	70%			
ERCP indication						
Malignant disease	27	6	81%	0.592		
Benign disease	101	23	81%			
BMI						
<18.5	49	7	88%	0.110		
≥18.5	79	22	79%			
ASA score						
≤2	91	19	83%	0.351		
≥3	37	10	79%			
Peritoneal dissemination						
Present	9	6	60%	0.035	0.021	4.71 (1.27-17.54)
Absent	119	23	84%			
Postoperative ileus						
Present	7	1	88%	0.546		
Absent	121	28	81%			
Splenectomy						
Present	23	7	77%	0.299		
Absent	105	22	83%			
Number of abdominal operations						
1 time	92	19	83%	0.320		
≥2 times	36	10	78%			
Surgical records						
Available	85	13	87%	0.027	0.245	
Not available	43	16	73%			
Endoscopists						
Trainer	55	15	79%	0.257		
Trainee	73	14	84%			
Passive bending section						
Equipped	106	21	83%	0.153		
Not equipped	22	8	73%			

ASA: American Society of Anesthesiologists; BMI: body mass index; CI: confidence interval; ERCP: endoscopic retrograde cholangiopancreatography; SD: standard deviation.

**Table 3:** Causes of failure to reach the blind end.

	With gastrectomy *N* = 15	Without gastrectomy *N* = 14	Total *N* = 29
Insufficient sSBE length	27% (4)	93% (13)	59% (17)
Malignant peritoneal stricture due to peritoneal dissemination	27% (4)	7% (1)	17% (5)
Others	46% (7)	0% (0)	24% (7)
Rate of reaching the blind end after switching to a long-type SBE in patients with an insufficient endoscope length	75% (3/4)	85% (11/13)	82% (14/17)

**Table 4:** Comparison of the rate of reaching the blind end: sSBE vs. sSBE+lSBE.

	sSBE	sSBE+lSBE	*p* value
R-Y with gastrectomy	88% (107/122)	90% (110/122)	0.342
R-Y without gastrectomy	60% (21/35)	91% (32/35)	0.002

R-Y: Roux-en-Y; SBE: single-balloon enteroscope; lSBE: long-type SBE; sSBE: short-type SBE.

## Data Availability

The data used to support the findings of this study are available from the corresponding author upon request.
